# Urinary Volatiles and Chemical Characterisation for the Non-Invasive Detection of Prostate and Bladder Cancers

**DOI:** 10.3390/bios11110437

**Published:** 2021-11-03

**Authors:** Heena Tyagi, Emma Daulton, Ayman S. Bannaga, Ramesh P. Arasaradnam, James A. Covington

**Affiliations:** 1 School of Engineering, University of Warwick, Coventry CV4 7AL, UK; Heena.Tyagi@warwick.ac.uk (H.T.); e.daulton@warwick.ac.uk (E.D.); 2 Department of Gastroenterology, University Hospital Coventry & Warwickshire, Coventry CV2 2DX, UK; ayman.bannaga@warwick.ac.uk (A.S.B.); r.arasaradnam@warwick.ac.uk (R.P.A.); 3 Warwick Medical School, University of Warwick, Coventry CV4 7HL, UK; 4 School of Health Sciences, Coventry University, Coventry CV1 5FB, UK; 5 School of Biological Sciences, University of Leicester, Leicester LE1 7RH, UK

**Keywords:** bladder cancer, prostate cancer, urinary biomarkers, urinary VOCs, machine olfaction, GC-IMS, GC-TOF-MS

## Abstract

Bladder cancer (BCa) and prostate cancer (PCa) are some of the most common cancers in the world. In both BCa and PCa, the diagnosis is often confirmed with an invasive technique that carries a risk to the patient. Consequently, a non-invasive diagnostic approach would be medically desirable and beneficial to the patient. The use of volatile organic compounds (VOCs) for disease diagnosis, including cancer, is a promising research area that could support the diagnosis process. In this study, we investigated the urinary VOC profiles in BCa, PCa patients and non-cancerous controls by using gas chromatography-ion mobility spectrometry (GC-IMS) and gas chromatography time-of-flight mass spectrometry (GC-TOF-MS) to analyse patient samples. GC-IMS separated BCa from PCa (area under the curve: AUC: 0.97 (0.93–1.00)), BCa vs. non-cancerous (AUC: 0.95 (0.90–0.99)) and PCa vs. non-cancerous (AUC: 0.89 (0.83–0.94)) whereas GC-TOF-MS differentiated BCa from PCa (AUC: 0.84 (0.73–0.93)), BCa vs. non-cancerous (AUC: 0.81 (0.70–0.90)) and PCa vs. non-cancerous (AUC: 0.94 (0.90–0.97)). According to our study, a total of 34 biomarkers were found using GC-TOF-MS data, of which 13 VOCs were associated with BCa, seven were associated with PCa, and 14 VOCs were found in the comparison of BCa and PCa.

## 1. Introduction

Early detection and diagnosis of cancer remains a key goal to improve the prognosis and life expectancy of patients [1,2,3,4]. Globally, cancer results in some of the highest mortality rates for any disease. In 2020 alone there were more than 19 million new cancer diagnoses and almost 10 million deaths [5]. The UK is a major contributor to this, with some of the highest cancer rates in the world. It is amongst the top 10% of countries, with the highest number of new cases of cancer [6]. These figures emphasize the importance of using screening methods to improve disease diagnosis and to reduce cancer morbidity [7].

Bladder cancer (BCa) is the ninth most common cancer worldwide and is also one of the most difficult cancers to diagnose and clinically manage [8,9]. Cystoscopy followed by transurethral resection of a bladder tumour (TURBT) with biopsy and histological assessments are considered to be the ‘Gold Standard’ for the diagnosis of BCa [10]. However, cystoscopy is invasive in nature, and can cause pain, urinary infections, and blood loss in some cases [11,12]. To aid in the diagnosis of BCa, a range of urine tests have been developed including the bladder tumour antigen (BTA) test, nuclear matrix protein 22 (NMP22), urinary bladder cancer antigen (UBC), and fibrin degradation products (FDP). Unfortunately, none of these tests have demonstrated sufficient specificity or sensitivity as a screening test [13].

Prostate cancer (PCa) occurs in men and is the sixth most common cancer worldwide [14,15,16]. For prostate cancer, PSA (prostate-specific antigen) is a commonly used blood test. However, it lacks sensitivity and specificity. PSA can be used for monitoring PC progression in both symptomatic and asymptomatic patients [17]. The downsides of PSA, as a diagnostic test for PCa patients, are mainly related to the high false-positive rate. PSA can be raised in urinary and prostate infections or other conditions such as benign prostatic hyperplasia (BHP) [18]. Therefore, a raised PSA level can lead to unnecessary biopsies, which may end up causing fever, pain, bleeding, and infection to the patient [19,20,21]. Recent European Association of Urology guidelines advise undertaking a multiparametric magnetic resonance scan on all patients prior to confirmatory biopsies; however, this is not always accessible, especially in low-resource settings [22].

One area receiving significant interest is in the use of volatile organic compounds (VOCs) to diagnose and monitor cancer. VOCs are chemical compounds that are either produced in vitro or are introduced externally and can indicate the presence, or absence, of disease in the body. The concept first emerged after reports indicated that dogs could recognise cancer by sniffing biological samples [23]. Since this discovery, researchers have reported that VOCs could be used to detect a broad range of cancers including lung, colorectal and pancreatic cancer [24,25,26,27].

Urine is a common biological source of VOCs, as the components present are either the intermediate products or end products of metabolic activities occurring inside the human body [28]. A study published in 2016 provided significant evidence for the use of urinary VOCs for distinguishing BCa, from a total of 72 urine samples the results showed an accuracy of 89%, 90% sensitivity, and 88% specificity using PLS-DA (partial least squares discriminant analysis) on GC-MS (gas chromatography-mass spectrometry) data [29].

Urine cytology is a non-invasive test which uses urine as biological modality for the presence of cancer. Several studies showed that though it exhibits high specificity, the sensitivity and specificity highly depend on collection method and cancer grade [30,31,32].

The gold standard for the analysis of VOCs remains GC-MS, but it is expensive, requiring specialised equipment and trained staff, making it difficult to implement in a point of care scenario. A variant of this is GC-TOF-MS (gas chromatography-time of flight-mass spectrometry), which is a similar technique used for multidimensional analysis of complex samples with the potential to identify an even greater number of VOCs [33,34]. However, more recently a range of other techniques have been reported that have the potential to be used at the point of care. GC-IMS (gas chromatography-ion mobility spectrometry) is one such technique, it provides high sensitivity and selectivity, and the GC-IMS can be created in a portable form factor and can use nitrogen or air as the carrier gas. However, it is less able to identify specific biomarkers and it is unable to identify chemicals with a low proton affinity. Our group has reported the use of this method with a range of different diseases [35,36]. Thus, the combination of GC-TOF-MS, which can provide a means of identifying specific biomarkers, with GC-IMS, a lower cost platform, using air as the carrier and thus facilitating ward use, is advantageous.

The study aimed to identify and test the potential of urinary biomarkers to distinguish between two different cancers and healthy controls using both GC-TOF-MS and GC-IMS. We believe this is the first time that GC-IMS has been used with these cancers in combination with GC-TOF-MS.

## 2. Materials and Methods

### 2.1. Urine Samples

A total of 106 patients were recruited after providing informed consent at University Hospital Coventry and Warwickshire NHS Trust, UK. Patients were recruited prior to anti-cancer treatment. This study was approved by Coventry and Warwickshire and North-East Yorkshire NHS Ethics Committees (Ref 18717 and Ref 260179). Urine samples were collected in standard universal sterile specimen containers and frozen within 2 h at −80 °C for subsequent batch analysis and according to standard operating procedures, compliant with tissue bank requirements under Human Tissue Act 2004. No chemicals were added to the urine before freezing, as we have previously shown that urine samples remain stable for extended periods of time at this temperature [37]. Prior to analysis the samples were transferred to the University of Warwick and briefly stored at −20 °C. The samples were defrosted in a laboratory fridge at 4 °C and aliquoted into 20 mL glass sample vials with a crimp cap. We used 5 mL of each urine sample for the analysis with GC-IMS and GC-TOF-MS. Of the 106 urine samples collected, 15 patients had confirmed BCa, 55 were confirmed PCa, and there were 36 non-cancerous controls. The mean age of the BCa patients was 70 years and the mean age of the PCa patients was 72 years. The demographic data of the subjects are illustrated in Table 1.

### 2.2. Analytical Devices

#### 2.2.1. G.A.S. FlavourSpec Gas Chromatography-Ion Mobility Spectrometry (GC-IMS)

The G.A.S FlavourSpec (Germany) uses a GC-IMS measurement technique to analyse VOCs. GC-IMS is a method used in various applications, such as detection of explosives and chemicals [40,41,42], air quality [43], health and disease detection [44,45,46] and food [47,48,49]. The method is formed of two stages. The first stage is a GC component that pre-separates chemicals based on their interaction with a retentive coating on the inside of a GC column. Thus, chemicals elude from the GC at different times [50]. These chemicals are further analysed using a drift-tube IMS method. Here, the chemicals are ionised (using a tritium source in our case) and pass along a drift-tube, propelled by a high electric field. Against the flow of ions, a buffer gas (using nitrogen in this case) is passed. The buffer gas and the ions collide resulting in a loss of momentum of the ions. Thus, the transit time along the tube is a function of the interaction of the ion with the electric field and the number of collisions with the buffer gas. This provides two-dimensional separation of the chemical components [48,51].

For analysis, glass vials containing samples were transferred to an autosampler fitted to the GC-IMS. The sample tray was chilled to 4 °C to reduce sample degradation during sample analysis. Each sample was heated to 40 °C and agitated for 10 min before sampling. The autosampler then took 0.5 mL of sample headspace and directly injected it into the GC-IMS. Urinary headspace was defined as the volume of gas above the urine sample inside the vial, which was in chemical equilibrium with liquid phase urine. The machine settings for analysis were as follows: E1: 150 mL/min (for the drift tube IMS), E2: 20 mL/min (for the GC column), and the pump was set to 25%. The total run time per sample was 10 min. The temperatures were set to T1 (IMS): 45 °C, T2 (column): 80 °C, and T3 (injector): 70 °C.

#### 2.2.2. Markes Gas Chromatography Time-of-Flight Mass Spectrometry (GC-TOF-MS)

GC-TOF-MS operates by analysing the time of flight of ions and analyse them according to their mass-to-charge ratio. The GC-TOF-MS system used was a combination of a TRACE 1300 GC (Thermo Fisher Scientific, Loughborough, UK) and a BenchTOF-HD TOF-MS (Markes Intl., Llantrisant, UK). This system also included a high-throughput autosampler and a thermal desorption unit, ULTRA-xr and UNITY-xr, respectively (both from Markes Intl.). The GC separated the chemicals in the same way as explained previously. The separated chemicals were detected by TOF MS once they entered the TOF ‘flight box’. TOF-MS separates fragment ions instead of molecular ions as in an IMS. The ions are detected depending upon the mass-to-charge ratio of the ions after passing through the drift tube [52,53].

For analysis, a thermal desorption (TD) sorbent tube (C2-AXXX-5149, Markes Intl., Llantrisant, UK) was inserted through the septum and into the headspace above the sample and then heated at 40 °C for 20 min. A pump was then attached to the TD tube, and whilst still being heated to 40 °C, the headspace VOCs were then pulled onto the tubes at 20 mL/minute for a further 20 min. The sorbent tubes were then placed in an autosampler for analysis. The analysis started with ULTRA-xr with a stand-by split set to 150 °C. The GC run time for samples was 25 min with a programmed temperature ramp from 40 °C to 280 °C at 20 °C/min. Each sample was pre-purged for 1 min and then desorbed at 250 °C for 10 min, with the trap purge time set to 1 min. These traps were then cooled at −30 °C and the trap was purged for 3 min at a temperature of 300 °C. The temperature for both transfer line and ion source were heated to 250 °C. The chemicals from GC-TOF-MS analysis were identified using the national institute of standards and technology (NIST) list (2011).

### 2.3. Statistical Methods

For GC-IMS data analysis, the data were extracted using the G.A.S VOCal (v0.1.3, G.A.S., Dortmund, Germany) software. This was followed by pre-processing steps to reduce the data’s dimensionality. Among all the data points, the central section contained all the computationally significant chemical information and thus all the other data were removed through a cropping process. This was followed by applying a small threshold to remove the background information, which was a value just above the background noise level. The same data cropping and threshold values were used on all the data, and it was undertaken using an automated program. The data were then analysed using a 10-fold cross-validation, undertaken using a bespoke R program (version 3.6.2). Within each fold training set, feature selection was undertaken using a Wilcoxon rank-sum test between the different cancer groups and non-cancerous group. That resulted in the identification of the 20 most discriminatory features between the two groups and the features trained by three models, XGBoost, logistic regression, and random forest. The model was then applied to the test set to create class probabilities. Once all the samples had been within a test set, statistical results were generated from the probabilities, including a receiver operator characteristic (ROC) curve, area under the curve (AUC), sensitivity, specificity, positive predictive value (PPV), and negative predictive value (NPV).

**Figure 1:**
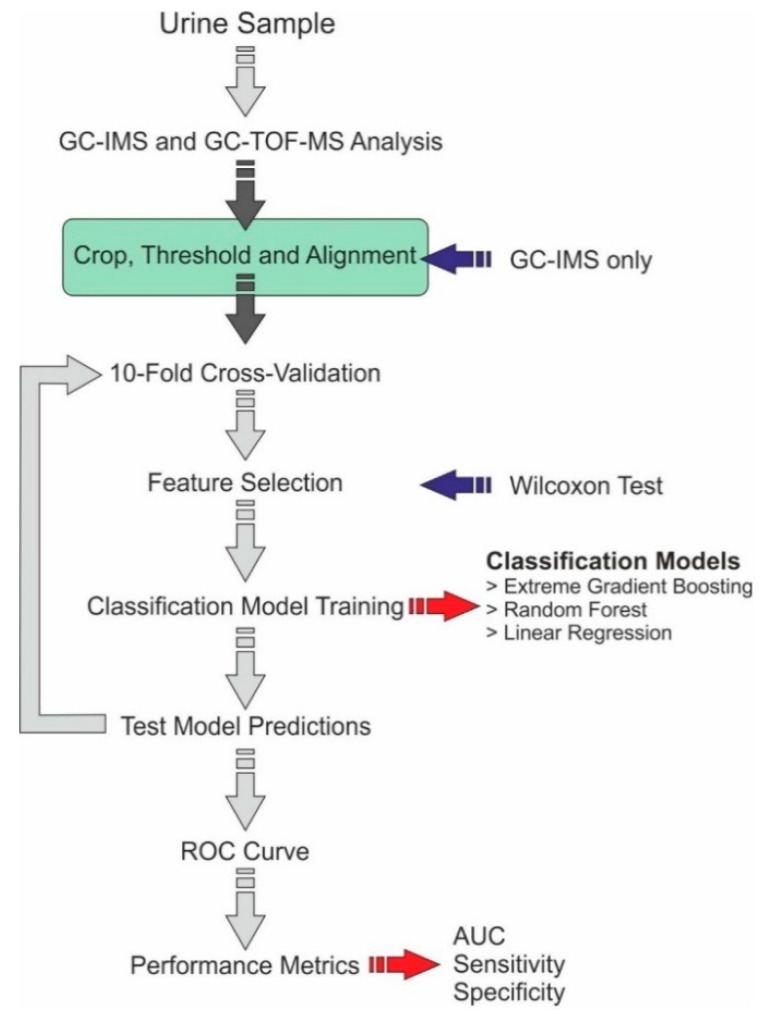
Data analysis pipeline.

An analogous approach was used for GC-TOF-MS data analysis. For GC-TOF-MS, the chemicals and the abundance of the chemicals were identified. Using the TOF-DS software, a background correction was applied, and the chromatogram was integrated, and the peaks were identified using the NIST list which was exported. The data obtained from GC-TOF-MS were converted into text files of chemical lists and abundances. The data were then processed using an ‘R’ program that was like that used for GC-IMS, where chemical components of discriminative power were identified. Figure 1 provides a flow diagram of the data analysis steps.

## 3. Results

**Figure 2:**
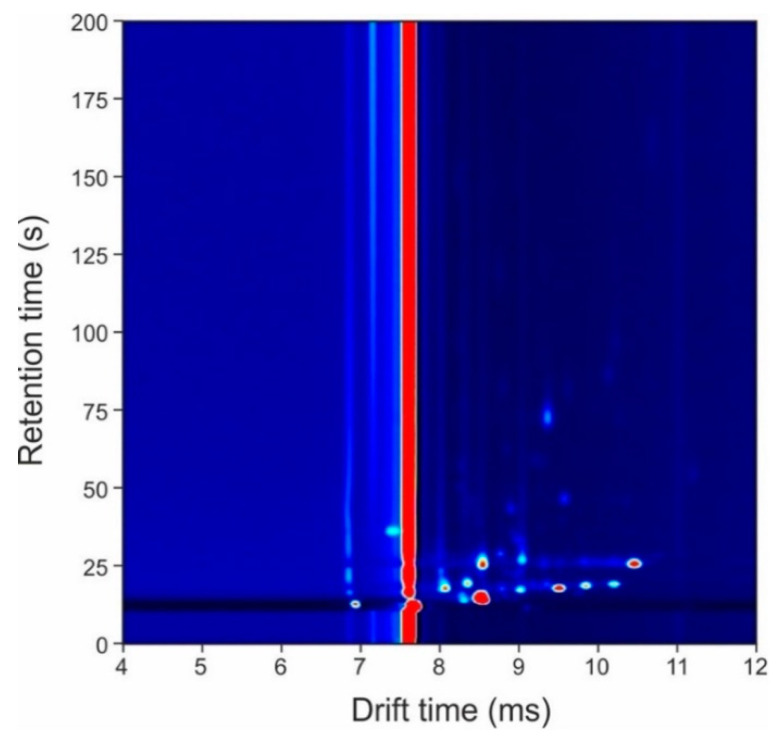
Typical output plot from the gas chromatography-ion mobility spectrometry (GC-IMS) instrument.

Figure 2 shows a typical output from the GC-IMS method from a urine sample in which the *x*-axis represents the drift time of the IMS and the *y*-axis represents the retention time of the GC. In the figure, the ‘dots’ are the chemicals detected by the IMS and the intensity of the peak represents the number of ions. Those ‘dots’ in red are the most intense. The red line in the figure is the default output of the instrument where no chemicals are present. G.A.S VOCal (v0.1.3, G.A.S., Dortmund, Germany) was used to view the GC-IMS data.

**Figure 3:**
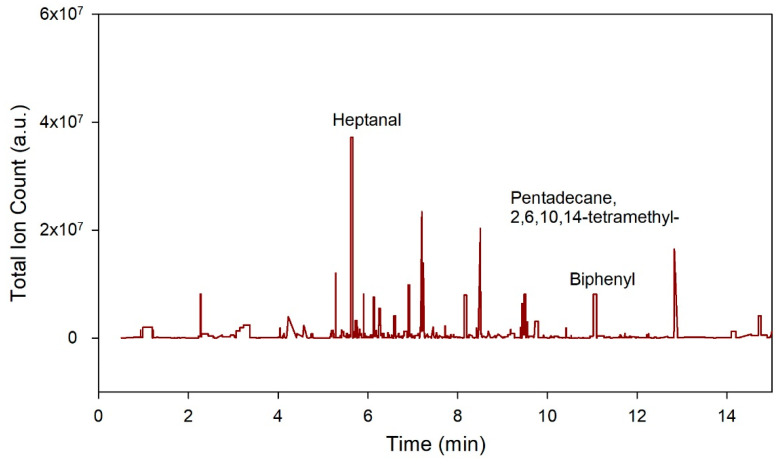
Figure illustrates a typical output plot of gas chromatography time-of-flight mass spectrometry (GC-TOF-MS). The *x*-axis in the plot represents the retention time and *y*-axis lists the chemical according to their abundance in the sample.

Figure 3 provides an example output from the GC-TOF-MS method. Here, the *x*-axis refers to the retention time, and the *y*-axis, the total ion count.

The results of the statistical analysis of the GC-IMS gathered results between different cancer groups and the non-cancerous group are given in Table 2. The results demonstrate high sensitivity and specificity, indicating that there are significant differences between the VOC profiles of the different groups. Importantly, good separation between the two different cancers, BCa vs. PCa, was also achieved. The false negative rate calculated for the GC-IMS analytical method in the study was 0.40 for BCa versus the PCa comparison, 0.13 for BCa versus the non-cancerous group and 0.24 for PCa versus the non-cancerous group, whereas the false positive rate was 0.02 for BCa versus the PCa group, 0.08 for BCa versus the non-cancerous group and 0.12 for PCa versus the non-cancerous group.

**Figure 4:**
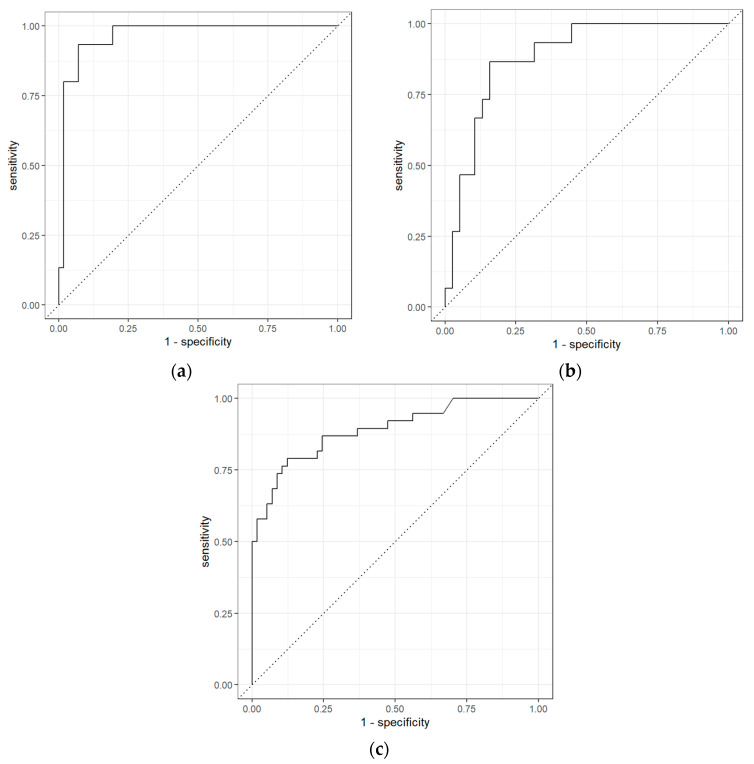
Receiver operator characteristic for (**a**) bladder cancer vs. PCa; (**b**) bladder cancer vs. non-cancerous group; and (**c**) prostate cancer vs. non-cancerous group using GC-IMS.

The ROC curves obtained from GC-IMS data comparing BCa and the non-cancerous group, BCa and PCa groups, and PCa and non-cancerous groups are shown in Figure 4. The results indicate that among BCa patients and PCa patients, AUC (area under the curve) was 0.97 (0.93–1.00) with sensitivity and specificity of 0.60 (0.38–0.80) and 0.98 (0.95–1.00), respectively. However, the separation between BCa and non-cancerous samples was even higher with a sensitivity of 0.87 (0.70–1.00), specificity of 0.92 (0.84–0.98) and AUC of 0.95 (0.90–0.99). Similarly, for PCa vs non-cancerous samples using GC-IMS, the separation was significant with a sensitivity of 0.76 (0.64–0.88), specificity of 0.88 (0.80–0.95) and AUC of 0.89 (0.83–0.94).

The results of the statistical analysis between different cancer groups for GC-TOF-IMS are given in Table 3. The results demonstrate high sensitivity and specificity, indicating that there are significant differences between the VOC profiles of different cancer groups, which was also shown in the GC-IMS data. The results showed that the false negative rate for BCa versus PCa comparison was 0.47, for BCa versus the non-cancerous group was 0.73 and for PCa versus the non-cancerous group was 0.22 for the GC-TOF-MS analytical method. The false positive rate for BCa versus PCa comparison was 0.1, for BCa versus the non-cancerous group it was 0.06, and for PCa versus the non-cancerous group it was 0.12.

**Figure 5:**
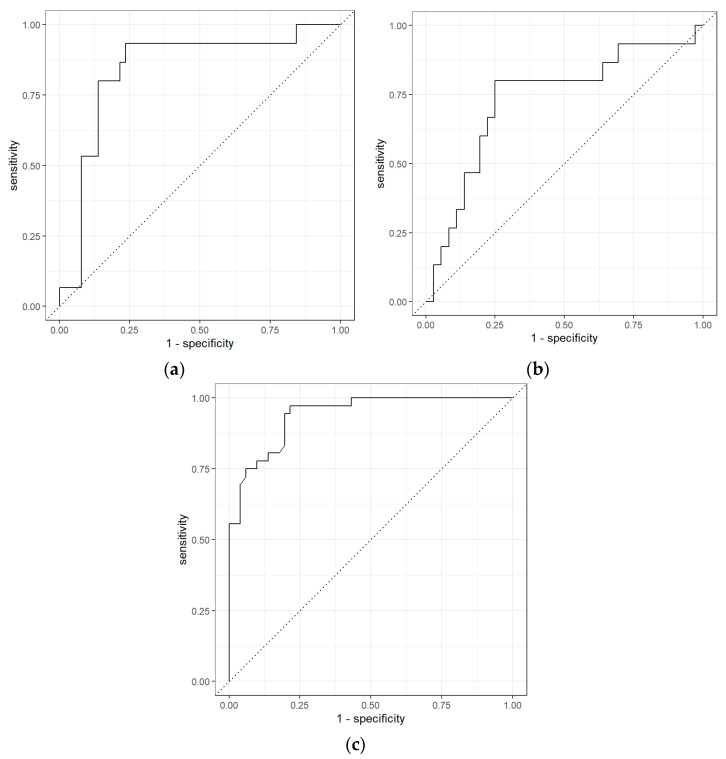
ROC for (**a**) bladder cancer vs. PCa; (**b**) bladder cancer vs. non-cancerous group; and (**c**) prostate cancer vs. non-cancerous group using GC-TOF-MS.

The ROC curves obtained from GC-TOF-MS data comparing BCa and non-cancerous groups, BCa and PCa groups, and PCa and non-cancerous groups are shown in Figure 5. The results indicate that GC-TOF-MS was able to differentiate BCa and PCa with AUC 0.84 (0.73–0.93), sensitivity and specificity of 0.53 (0.33–0.75) and 0.90 (0.83–0.96). The separation between BCa and non-cancerous samples was very poor with sensitivity only 0.27 (0.9–0.46), specificity 0.94 (0.88–1.00) and AUC 0.82 (0.72–0.90). However, the separation was more significant with sensitivity 0.78 (0.66–0.89), specificity 0.88 (0.80–0.95) and AUC 0.94 (0.90–0.97) for PCa and non-cancerous groups.

In our results, we analysed different VOCs linked to BCa and PCa for the screening and diagnosis of these cancers. A total of 34 biomarkers were found using TOF-DS software. These VOCs were verified using PubChem, NIST (National Institute of Standards and Technology), and previously published papers. Out of 34, 13 VOCs were found in the comparison of BCa and non-cancerous groups specific to BCa, as shown in Table 4, seven in PCa and non-cancerous groups specific to PCa, as shown in Table 5, and 14 VOCs were found in the comparison of BCa and PCa group, as shown in Table 6, out of which 3 VOCs do not overlap either with BCa or PCa, which may indicate that they are new markers.

## 4. Discussion

In our study, we found that both GC-IMS and GC-TOF-MS were able to separate different cancer groups from each other as well as non-cancerous group. The separation between BCa from non-cancerous group was highest using GC-IMS with 0.95 AUC (0.87 sensitivity and 0.92 specificity). A similar study conducted by Weber et al. [54] suggested overall accuracy of 70% (70% sensitivity and 70% specificity) using urinary headspace for the analysis of BCa using gas sensors. Another study conducted by Khalid et al. [55] showed very high statistical results using an in-house GC-sensor device. They used two models for analysis suggesting 100% sensitivity and 94.6% specificity using a linear discriminant analysis (LDA) model and 95.8% sensitivity and 94.6% specificity using PLS-DA.

The separation between PCa and the non-cancerous group was highest using GC-TOF-MS method with 0.94 AUC (0.78 sensitivity and 0.88 specificity) whereas the study conducted by Gao et al. [56] for the analysis of urinary VOCs for prostate cancer calculated 0.92 AUC (0.96 sensitivity and 0.80 specificity). Another study conducted by Lima et al. [57] used PLS-DA to discriminate PCa from non-cancerous group with an AUC of 0.83 (84% sensitivity and 80% specificity) using urine headspace.

In this study, we developed urinary VOC profiles linked with BCa and PCa. Table 4 consists of the chemicals that have been identified in our study and have been cross verified using PubChem, NIST and previously published research, which may have relevance to BCa diagnosis.

Out of 13 VOCs found to be noteworthy to BCa, biphenyl, heptanal, and 2,6,10,14-tetramethyl-pentadecane were the three distinct biomarkers found in our study that did not overlap with other studies. Biphenyl has been identified as the most significant biomarker in our study. Biphenyl has been linked to various diseases, including carcinoma. It has been proven that biphenyl is a promoter of BCa in rats [58]. Biphenyl has been found to be metabolized in the liver [59]. Heptanal is reported to present in the blood of lung cancer patients [60]. According to the HMBD (Human Metabolome Database), the biological activity of heptanal inside humans can cause digestive disorder including associated with the bladder [61]. 2,6,10,14-tetramethyl-pentadecane is reported as carcinogens but is mentioned far less in the literature [62]. Nonanal, tetradecane, dodecane, hexadecane, naphthalene, and methyl isobutyl ketone were suggested by Rodrigues et al. [63] in their study using GC-MS on BCa cell lines whereas 2-pentanone and 4-heptanone overlap with the findings of Cauchi et al. [29]. Benzoic acid was another chemical found in our study that overlapped in both Rodrigues et al. [63] and Cauchi et al. [29].

From the analysis of PCa urine samples, a total of seven distinct VOCs were identified and are summarised in Table 5. In our study, we found toluene as the most significant chemical for PCa. Toluene has been published previously as a significant biomarker for PCa [64]. In addition, it has been reported that toluene has been found to be associated with testicular diseases [65,66]. Pyrrole has been reported by Smith et al. in their study with 24 controls and 13 patients with PCa. They tested the urine samples to assess VOC profiles and found pyrrole to be one of the significant markers for PCa [67]. 2-Ethyl-1-hexanol, phenol and dimethyl disulphide [68], acetic acid [69], and 2-methyl cyclopentanone [70] were also found in our study, which overlaps with previous studies.

Table 6 represents all the chemicals found in the analysis of urine samples for prostate versus BCa. Most of the chemicals present in this list are like those found in Table 4 and Table 5. 2-Hexanone, p-xylene, and 3-methyl nonane are the only significant chemicals out of 14 in this list that are important for separating BCa and PCa. 2-Hexanone and p-xylene have previously been reported as significant markers for the PCa [68,70]. There is no significant evidence for both 2-hexanone and p-xylene as a potential biomarker for BCa. However, 3-methyl-Nonane has not yet been reported as a biomarker for either bladder or PCa, although they have been reported as a biomarker for lung cancer in different studies [71,72]. This may signify the importance of 3-methyl-nonane as a potentially significant marker. The results reported in this paper support the findings of other groups for the validation of these chemicals as potential biomarkers in both PCa and BCa. It has been noted that the chemicals found in all the cancer groups were different and there was almost no overlapping of the VOC fingerprints for BCa and PCa. This adds further support to the unique VOC fingerprint in cancers of different cell origins [73].

The use of urinary VOC analysis is an attractive option due to the non-invasive nature. It also has the potential to be used in early cancer diagnosis with further validation studies. This approach may also prove to be efficient, whilst lowering the cost per patient, and increasing patient compliance due to its non-invasive nature. The results of using GC-IMS as an analysis tool are significant as the method is much simpler than using a high-end analytical method, such as GC-MS, and without the need for a laboratory environment. We believe that using VOCs to analyse human waste will be an important diagnostic tool for the future. Cancer may well be one area of focus and may be used as part of the UK 2-week wait screening program to help reduce the number of unneeded procedures. The key is to run more larger studies targeting these cancers and to have tools that are CE marked (or equivalent) for cancer diagnosis. We plan to use urine VOCs in association with other tests in future which help to improve the performance and achieve a more in depth understanding of VOCs and their metabolic pathways.

Our results were limited by not accounting for the contributory factors that can also lead to abnormal metabolism with subsequent excretion of differing concentrations of these chemicals in the urine. These factors include stress, alcohol, smoking, certain food products, medicines, and different environmental factors. Several studies have reported the effect of smoking on VOCs [74,75]. Study conducted by A. McWilliams et al. showed that active smoking had an impact on urinary VOC profiles associated with current smokers and ex-smokers [76]. We aim to consider these further in the next study. We also did not undertake full chemical identification with calibration standards. However, many of the chemicals we found correlate with other studies and, therefore, there is evidence that these are correct.

## 5. Conclusions

In this paper, GC-IMS and GC-TOF-MS methods were used to identify VOC fingerprints using urine headspace and establish an interdependence between BCa, PCa and non-cancerous samples. It was found that both GC-IMS and GC-TOF-MS have the potential to differentiate between different cancer groups with respective AUC for different diagnostic groups: For GC-IMS, BCa and PCa (0.97 (0.93–1)), BCa and non-cancerous (0.95 (0.90–0.99)), PCa and non-cancerous (0.89 (0.83–0.94)) and for GC-TOF-MS, BCa and PCa (0.84 (0.73–0.93)), BCa and non-cancerous (0.81(0.70–0.90)), PCa and non-cancerous (0.94 (0.90–0.97)). A total of 35 VOCs were found to be relevant for identifying these cancer groups, with several VOCs distinct to each cancer. VOCs from this study were supported by findings from previous studies. This signifies that VOCs for both bladder and prostate cancer have different profiles, which may be helpful in future to distinguish them. In the future, the VOC profiles obtained from these analytical devices can be used as a reference for developing low-cost devices. It is plausible that VOC profiles can be used as an adjunct to diagnosis enabling selection of only high-risk groups to undergo cystoscopy examinations which will be widely beneficial considering limited capacity and cost.

## Figures and Tables

**Table 1:** Demographic data for subject groups.

Group	Bladder Cancer	Prostate Cancer	Non-Cancerous
Number of samples	15	55	36
Mean Age (years)	70.0	71.9	62.5
Sex: Male/Female	12:3	All Male	24:12
Mean BMI (Kg/m^2)^	24.4	27.5	30.9
Current Smoker n (% of patients)	1 (6.7%)	6 (10.9%)	3 (8.3%)
Mean PSA level (ng/mL)	-	20.6 (3.6–153.90)	-
Gleason score	-	Case 01 4 + 5 = 9 Case 02 3 + 4 = 7 Case 03 3 + 3 = 6 Case 04 4 + 5 = 9 Case 05 4 + 5 = 9 Case 06 3 + 4 = 7 Case 07 3 + 4 = 7 Case 08 3 + 5 = 8 Case 09 5 + 4 = 9 Case 10 3 + 4 = 7 Case 11 3 + 3 = 6 Case 12 3 + 4 = 7 Case 13 3 + 3 = 6 Case 14 4 + 5 = 9 Case 15 3 + 4 = 7 Case 16 3 + 4 = 7 Case 17 3 + 4 = 7 Case 18 3 + 4 = 7 Case 19 3 + 4 = 7 Case 20 3 + 3 = 6 Case 21 4 + 5 = 9 Case 22 3 + 3 = 6 Case 23 4 + 3 = 7 Case 24 3 + 4 = 7 Case 25 4 + 4 = 8 Case 26 3 + 3 = 6 Case 27 4 + 5 = 9 Case 28 4 + 4 = 8 Case 29 3 + 3 = 6 Case 30 3 + 3 = 6 Case 31 4 + 4 = 8 Case 32 3 + 4 = 7 Case 33 4 + 5 = 9 Case 34 3 + 4 = 7 Case 35 3 + 4 = 7 Case 36 3 + 4 = 7 Case 37 3 + 4 = 7 Case 38 3 + 5 = 8 Case 39 4 + 5 = 9 Case 40 3 + 4 = 7 Case 41 3 + 4 = 7 Case 42 3 + 4 = 7 Case 43 3 + 5 = 8 Case 44 3 + 4 = 7 Case 45 5 + 5 = 10 Case 46 4 + 5 = 9 Case 47 4 + 4 = 8 Case 48 3 + 4 = 7 Case 49 4 + 3 = 7 Case 50 3 + 3 = 6 Case 51 4 + 5 = 9 Case 52 4 + 4 + 8 Case 53 3 + 3 = 6 Case 54 3 + 4 = 7 Case 55 3 + 3 = 6	-
WHO 1973 Grade	Case 01 G2 Case 02 G3 Case 03 G3 Case 04 G1 Case 05 G2 Case 06 G3 Case 07 G1 Case 08 G3 Case 09 G3 Case 10 G1 Case 11 G3 Case 12 G1 Case 13 G1 Case 14 G2 Case 15 G1	-	-
Prostate cancer Gleason grading: Score ≤ 6, pattern ≤ 3 + 3. This refers to Grade 1. Tumour cells look like normal prostate cells with only individual discrete well-formed glands. Score 7, pattern 3 + 4. This refers to Grade 2. Tumour with well-form glands and lesser component of poorly differentiated glands. Score 7, pattern 4 + 3. This refers to Grade 3. Tumour has predominantly poorly formed/fused/cribriform glands with lesser component of well-formed glands. Score 8, pattern 4 + 4, 3 + 5 and 5 + 3. This refers to Grade 4. Tumour with only poorly formed/fused/cribriform glands. Score 9 or 10, pattern 4 + 5, 5 + 4 and 5 + 5. This refers to Grade 5. Tumour lacking gland formation (or with necrosis) with or without poorly formed/fused/cribriform glands [38]. G1 low grade differentiation, G2 moderate grade differentiation and G3 is high grade differentiation [39].			

**Table 2:** GC-IMS diagnostic group results.

Comparisons	Classifiers	AUC	Sensitivity	Specificity	PPV	NPV
BCa vs. PCa	Logistic Regression with Elastic Net Regularization	0.97 (0.93–1.00)	0.60 (0.38–0.80)	0.98 (0.95–1.00)	0.90	0.90
BCa vs. non-Cancerous	Logistic Regression with Elastic Net Regularization	0.95 (0.90–0.99)	0.87 (0.70–1.00)	0.92 (0.84–0.98)	0.81	0.95
PCa vs. non-Cancerous	Extreme Gradient Boosting	0.89 (0.83–0.94)	0.76 (0.64–0.88)	0.88 (0.80–0.95)	0.81	0.85

**Table 3:** GC-TOF-MS diagnostic group results.

Comparisons	Classifiers	AUC	Sensitivity	Specificity	PPV	NPV
BCa vs. PCa	Logistic Regression with Elastic Net Regularization	0.84 (0.73–0.93)	0.53 (0.33–0.75)	0.90 (0.83–0.96)	0.62	0.87
BCa vs. non-Cancerous	Random Forest	0.81 (0.70–0.90)	0.27 (0.09–0.46)	0.94 (0.88–1.00)	0.33	0.71
PCa vs. Non-Cancerous	Random Forest	0.94 (0.90–0.97)	0.78 (0.66–0.89)	0.88 (0.80–0.95)	0.82	0.85

**Table 4:** A list of possible biomarkers from the analysis of urine samples by GC-TOF-MS identified using PubChem, NIST and publications significant to bladder cancer.

	Chemicals	*p*-Values	Molecular Weight (g/mol)
1	Biphenyl	<0.01	154.21
2	Nonanal	<0.01	142.24
3	Tetradecane	<0.01	198.39
4	Pentadecane, 2,6,10,14-tetramethyl-	0.012	268.5
5	2-Pentanone	0.012	86.13
6	Undecane	0.014	156.31
7	4-Heptanone	0.018	114.19
8	Dodecane	0.025	170.33
9	Hexadecane	0.026	226.44
10	Heptanal	0.026	114.19
11	Methyl Isobutyl Ketone	0.045	100.16
12	Naphthalene	0.046	128.169
13	Benzoic acid	0.049	122.12

**Table 5:** List of possible biomarkers from the analysis of urine samples by GC-TOF-MS identified using PubChem, NIST and publications significant to PCa.

	Chemicals	*p*-Values	Molecular Weight (g/mol)
1	Toluene	<0.01	92.14
2	Phenol	<0.01	325.4
3	Acetic acid	<0.01	60.05
4	1-Hexanol, 2-ethyl-	0.011	130.229
5	Disulfide, dimethyl	0.012	94.2
6	Cyclopentanone, 2-methyl-	0.017	98.14
7	Pyrrole	0.033	67.09

**Table 6:** List of possible biomarkers from the analysis of urine samples by GC-TOF-MS identified using PubChem, NIST and publications significant to PCa and bladder cancer.

	Chemicals	*p*-Values	Molecular Weight (g/mol)
1	Toluene	<0.01	92.14
2	Methyl Isobutyl Ketone	<0.01	100.16
3	Dodecane	<0.01	170.33
4	Phenol	<0.01	325.4
5	Cyclopentanone, 2-methyl-	<0.01	98.14
6	2-Hexanone	<0.01	100.16
7	Heptanal	<0.01	114.19
8	p-Xylene	<0.01	106.16
9	Nonane, 3-methyl-	<0.01	142.28
10	Tetradecane	<0.01	198.39
11	Nonanal	<0.01	142.24
12	Biphenyl	0.019	154.21
13	Acetic acid	0.025	60.05
14	2-Pentanone	0.032	86.13

## Data Availability

The data presented in this study are available on request from the corresponding author. The data are not publicly available due to the ethical approval.
